# Trendy, but Are They Safe? Current Popular Diets and Their Use in Inflammatory Bowel Diseases: Narrative Review

**DOI:** 10.3390/nu18193133

**Published:** 2026-09-23

**Authors:** Klara Szwarc, Kacper Kamil Zagaja, Alicja Ratajczak-Pawłowska, Dagmara Mahadea, Agnieszka Dobrowolska, Liliana Łykowska-Szuber

**Affiliations:** 1 Department of Gastroenterology, Dietetics and Internal Medicine, Poznan University of Medical Sciences, Poland, Przybyszewskiego 49, 60-355 Poznan, Poland; 2 Laboratory of Nutrigenetics, Department of Gastroenterology, Dietetics and Internal Diseases, Poznan University of Medical Sciences, 61-701 Poznan, Poland

**Keywords:** inflammatory bowel disease, Crohn’s disease, ulcerative colitis, Mediterranean diet, Western diet, ultra-processed foods, dietary patterns, nutritional therapy, food insecurity, gut microbiota

## Abstract

Inflammatory bowel disease (IBD) arises from the interplay between genetic susceptibility, immune dysregulation, microbiota, and environmental exposures, with diet potentially contributing to both pathogenesis and management. Patients frequently modify their diet independently, often using different strategies during remission and active symptoms. This narrative review synthesises evidence on patient-accessible dietary patterns and selected structured nutritional therapies across five domains: incident IBD, objective intestinal inflammation, gastrointestinal symptoms and quality of life, nutritional safety, and real-world feasibility. Mediterranean and other minimally processed plant-forward patterns are associated with higher diet quality and potentially favourable microbial and metabolic profiles; however, current trials do not demonstrate consistent superiority for objective remission or mucosal healing. Western and ultra-processed food-rich patterns are associated with incident IBD, whereas evidence for relapse in established disease is less consistent. Low-FODMAP approaches may improve selected symptoms but should not be interpreted as anti-inflammatory therapy. Evidence for ketogenic and intentional carnivore diets remains insufficient for routine use. Exclusive enteral nutrition and the Crohn’s Disease Exclusion Diet have distinct, protocolised therapeutic roles, particularly in Crohn’s disease. Across strategies, affordability, food security, cooking resources, disease phenotype, nutritional risk, and access to specialist dietetic care determine safe and equitable implementation.

## 1. Introduction

Inflammatory bowel disease (IBD), comprising Crohn’s disease (CD) and ulcerative colitis (UC), is a chronic immune-mediated condition with substantial effects on nutritional status and quality of life. Current pharmacological management includes corticosteroids, conventional immunomodulators, biological therapies targeting different inflammatory pathways, and increasingly small-molecule agents [1,2]. Although these therapies have substantially improved disease control, primary non-response or secondary loss of response, adverse effects, infection risk, treatment discontinuation, and treatment-specific monitoring requirements may limit their use [1,2,3]. In addition, contemporary IBD management carries a considerable healthcare and economic burden [4]. Nevertheless, sustained disease control is not achieved in all patients. Nutritional inadequacy is also common: in a European cohort of adults with CD, patients reported lower intakes of fibre and several micronutrients than healthy controls [5]. Experimental evidence further supports a potential role of diet in intestinal inflammation [6]. Consequently, dietary management has attracted increasing attention as a potentially modifiable and complementary component of IBD care.

IBD pathogenesis reflects interactions among genetic susceptibility, dysregulated immune responses, impaired epithelial barrier function, the intestinal microbiota, and environmental exposures. Diet may influence microbial substrates, bile acid metabolism, mucus integrity, epithelial permeability, and immune signalling [7,8]. CD and UC also differ substantially in anatomical distribution and clinical phenotype. UC is confined to the colon, whereas CD may affect any segment of the gastrointestinal tract and may develop stricturing or penetrating complications. Accordingly, disease location, inflammatory activity, strictures, previous intestinal surgery, and stoma status may influence dietary tolerance, nutritional requirements, and the appropriateness or safety of specific dietary interventions.

In the clinical context, dietary modifications in IBD may serve several distinct purposes. First, they may aim to modulate inflammatory activity through effects on immune and oxidative pathways, the gut microbiota, or the intestinal barrier. Second, they may alleviate gastrointestinal symptoms through restriction of individually poorly tolerated foods, without necessarily affecting inflammatory activity. Third, dietary modifications may help prevent or correct deficiencies in energy, protein, vitamins, minerals, and other nutrients. Therefore, symptom improvement should not automatically be interpreted as evidence of inflammatory control, and the effectiveness of dietary interventions should be assessed against their specific therapeutic objective [9].

This distinction matters when interpreting evidence on diet’s role in preventing IBD and treating established disease. General dietary patterns, such as the Mediterranean diet (MD), differ from disease-specific, protocolised therapies, such as exclusive enteral nutrition (EEN) and the Crohn’s disease exclusion diet (CDED) [9].

Diet is also highly relevant from the patient’s perspective. Patients frequently associate particular foods with gastrointestinal symptoms or disease exacerbations and often modify their diet without professional support. A 2026 scoping review showed that dietary modification after an IBD diagnosis is widespread and is driven by perceived symptom triggers, food avoidance, uncertainty, and limited access to reliable advice [10]. Nutritional knowledge remains variable, and patients often rely on online and non-specialist sources [11]. At the same time, modern treatment allows many patients to remain in prolonged remission. Their dietary choices may therefore reflect goals beyond control of inflammation, including symptom reduction, weight management, general health, convenience, work, travel, and lifestyle preferences.

This makes the safety and appropriateness of commonly available dietary patterns particularly important. Restrictive diets may improve selected symptoms but can also reduce dietary diversity and increase the risk of nutritional deficiencies. This is especially relevant because malnutrition, micronutrient deficiencies, and avoidant eating behaviours are already common in IBD [5,10,11]. The recent European Crohn’s and Colitis Organisation (ECCO) consensus recognises the role of diet in nutritional optimisation, symptom management, selected therapeutic settings, and possibly disease prevention, while emphasising substantial gaps in the evidence [9]. Disease phenotype must also be considered. For example, high-fibre approaches may require modification in clinically significant stricturing CD. Patients after intestinal resection or with a stoma may also have specific fluid, electrolyte, energy, and nutrient requirements [9].

Another increasingly recognised issue is the presence of irritable bowel syndrome (IBS)-like symptoms in patients with IBD, including in those with objectively quiescent disease. Persistent abdominal pain, bloating, urgency, or altered stool frequency may therefore reflect functional symptoms rather than active intestinal inflammation. In such patients, a low-FODMAP diet may help control symptoms [9]. More broadly, dietary factors associated with IBD development should not automatically be considered therapeutic once the disease is established, as prevention-stage and treatment-stage dietary exposures address fundamentally different clinical questions.

The literature describes the high efficacy of various nutritional interventions, such as the CDED, and their potential impact on the gut microbiome and IBD management [12]. However, in routine clinical practice, patients often choose or modify their dietary patterns independently. Their motivations may include improving well-being, reducing gastrointestinal symptoms, limiting the perceived risk of disease exacerbation, and, in some cases, achieving or maintaining clinical remission. These decisions may be based on individual observations of food tolerance, personal beliefs, or information from non-professional sources, while restrictive and elimination practices may also occur during clinical remission [10,11]. Dietary choices are also influenced by disease phenotype and activity, nutritional status, cultural factors, and access to professional dietary counselling [9,10,11]. This narrative review aims to critically assess the safety of popular dietary patterns used by patients with IBD, including their potential effects on gastrointestinal symptoms and inflammation, the risk of nutritional deficiencies, and nutritional status. We discuss formal IBD-specific dietary interventions only to distinguish structured nutritional therapies from dietary patterns patients adopt independently.

## 2. Methods

### 2.1. Review Design and Scope

This narrative review used a structured literature search to ensure broad, transparent identification of publications and an integrated, clinically oriented synthesis, rather than a formal assessment of pooled treatment effects. The review focused primarily on popular dietary patterns used in the general population that patients with IBD may adopt independently or follow in accordance with general dietary recommendations. The included dietary patterns were based on commonly available foods and were considered feasible for implementation in routine clinical practice.

The analysis focused on the potential impact of these dietary patterns on the course of IBD, including disease activity and intestinal inflammation, gastrointestinal symptoms and quality of life, as well as nutritional adequacy, safety, and practical feasibility. We discussed established IBD-specific nutritional therapies only as needed to distinguish protocol-based therapeutic interventions from dietary patterns independently adopted by patients.

### 2.2. Information Sources and Search Strategy

PubMed/MEDLINE, Scopus, and Web of Science were searched from database inception to 10 August 2026. Google Scholar was used as a supplementary source to identify additional relevant publications and recently published articles. Reference lists of relevant systematic reviews, meta-analyses, consensus statements, professional guidance, and key primary studies were also screened manually. Searches combined terms relating to IBD (“inflammatory bowel disease”, “Crohn disease”, “Crohn’s disease”, and “ulcerative colitis”) with terms describing dietary patterns and behaviours. Dietary terms included “Mediterranean diet”, “Western diet”, “plant-based diet”, “vegetarian diet”, “semi-vegetarian diet”, “low FODMAP”, “ketogenic diet”, “carnivore diet”, “ultra-processed food”, “Dietary Inflammatory Index”, “anti-inflammatory diet”, “pro-inflammatory diet”, “food avoidance”, and “dietary beliefs”. Separate searches addressed feasibility and equity using terms including “food insecurity”, “dietary cost”, “affordability”, “food access”, and “socioeconomic”. Searches concerning structured nutritional therapies included “exclusive enteral nutrition”, “Crohn’s Disease Exclusion Diet”, and “partial enteral nutrition”. An example PubMed search string was: (“inflammatory bowel disease” OR “Crohn disease” OR “Crohn’s disease” OR “ulcerative colitis”) AND (“Mediterranean diet” OR “Western diet” OR “plant-based diet” OR “low FODMAP” OR “ketogenic diet” OR “carnivore diet” OR “ultra-processed food” OR “exclusive enteral nutrition” OR “Crohn’s Disease Exclusion Diet”). Search syntax was adapted for Scopus and Web of Science as required.

### 2.3. Eligibility and Evidence Prioritisation

Human studies evaluating dietary patterns in relation to IBD development, disease activity, relapses, gastrointestinal symptoms, quality of life, nutritional outcomes, dietary behaviours, or feasibility were considered. Randomised controlled trials, prospective cohort studies, systematic reviews, meta-analyses, and current professional guidance were prioritised. Cross-sectional, case–control, qualitative, and survey-based studies were included when they addressed clinically relevant associations, patient behaviour, food avoidance, adherence, affordability, food insecurity, or access to nutritional care that was not captured by higher-level evidence. Publications in English were considered. Earlier landmark studies were retained when they remained the principal or only direct evidence for a specific dietary approach, while particular emphasis was placed on evidence published from 2021 onwards.

Animal and in vitro studies were used only to illustrate plausible biological mechanisms and were not treated as evidence of clinical efficacy. Studies focusing exclusively on isolated supplements or individual nutrients were generally outside the scope unless they directly informed the interpretation of a broader dietary pattern. Evidence was considered across five prespecified domains: (1) incident IBD; (2) objective inflammatory activity; (3) patient-reported gastrointestinal symptoms and quality of life; (4) nutritional adequacy and safety; and (5) feasibility. Objective inflammatory outcomes included faecal calprotectin, C-reactive protein, and endoscopic or histological activity, while feasibility encompassed adherence, cost, food security, access, acceptability, and treatment burden.

### 2.4. Study Selection and Narrative Synthesis

Two designated authors conducted the literature searches and assessed potentially eligible publications within predefined thematic sections. Approximately 200 records were assessed at the title and abstract level, and approximately 135 full-text articles were reviewed for potential inclusion. When eligibility or interpretation was uncertain, the two authors discussed the publication and reached consensus, consulting a senior author when necessary. As this work was designed as an integrative narrative review rather than a systematic review, records were not routinely screened independently in duplicate and no formal risk-of-bias assessment or certainty-of-evidence grading was performed. No meta-analysis was undertaken. The evidence was synthesised narratively, with greater interpretative weight given to studies with more robust designs, larger sample sizes, prospective rather than retrospective assessment, longer follow-up, and objective measures of intestinal inflammation. Consistency of findings across studies was also considered. Particular attention was paid to distinguishing symptomatic improvement from objectively demonstrated control of intestinal inflammation. Findings from observational and mechanistic studies were therefore interpreted as supportive or hypothesis-generating evidence and were not considered equivalent to evidence derived from randomised clinical trials.

## 3. A Patient-Centred Framework: Real-World Dietary Choices, Disease Activity, and Access

### 3.1. Why Patient-Accessible Dietary Patterns Matter

Most patients do not first encounter diet as a formal medical intervention. Instead, they modify familiar foods, remove perceived triggers, follow advice from social media or peer communities, or adopt named diets they can implement without specialised formula products. In this review, a “patient-accessible dietary pattern” is defined as an approach that can be followed using commonly available foods outside a research protocol. This includes Mediterranean, plant-forward or vegetarian, Western or UPF-rich, low-FODMAP, ketogenic, and carnivore approaches. Patient accessibility should not be conflated with high prevalence, affordability, nutritional adequacy, or therapeutic efficacy.

Dietary choices also change over time. During active symptoms, patients commonly reduce total intake, fibre, dairy products, spicy foods, fat, or entire food groups to reduce stool frequency, pain, and urgency. In remission, some restrictions persist because of fear of relapse even when objective inflammation is absent [10]. A patient-centred assessment must therefore distinguish diets selected to reduce immediate symptoms from patterns intended to modify long-term inflammatory activity. It must also consider whether the patient is in biochemical remission, has persistent functional symptoms, or has a phenotype such as a stricture or high-output stoma that genuinely changes dietary tolerance.

### 3.2. Affordability, Food Security, and Structural Barriers

Income, local food prices, food availability, transport, time, cooking facilities, health literacy, and access to an IBD-specialist dietitian shape the ability to follow a recommended diet. Food insecurity is particularly relevant because it can increase reliance on shelf-stable, inexpensive UPFs and reduce the capacity to purchase or prepare nutrient-dense foods that are tolerated. In a nutrition-focused IBD cohort, food insecurity was associated with poorer dietary quality and greater UPF intake [13]. A nationwide US analysis subsequently found that food insecurity remained more common among adults with IBD than among those without IBD and was strongly associated with social and insurance-related disadvantage [14].

The transferability of predominantly US- and UK-derived equity data to continental Europe, particularly Central and Eastern Europe (CEE), requires caution. The affordability of a healthy diet varies considerably across European countries; recent estimates based on 26 EU Member States showed marked differences in the proportion of household income required to meet healthy-diet costs, with a relatively greater burden in some lower-income countries, including Poland [15]. In adults with IBD, a 12-country European study, including several CEE countries, also demonstrated substantial between-country differences in out-of-pocket expenditures, including costs related to special diets and nutritional products [16]. Access to specialised nutritional care is similarly heterogeneous: the ECCO E-QUALITY survey found that only 59% of participating European centres had a dietitian or nutritionist with a specific interest in IBD. In contrast, nutritional assessment was reported less frequently in Eastern than in Southern or Western European centres [17]. Data on reimbursement of exclusive enteral nutrition (EEN) and partial enteral nutrition (PEN) are more limited in adults. Paediatric European data suggest that greater reimbursement and better dietitian availability are associated with more frequent EEN use [18]. However, these findings should not be directly extrapolated to adult IBD populations. Overall, CEE-specific adult data on food insecurity, dietary affordability, reimbursement of nutritional therapies, and access to IBD-specialist dietetic care remain limited.

Affordability should therefore be treated as a component of intervention feasibility and external validity. A biologically plausible recommendation that is financially or logistically inaccessible is unlikely to be sustained and may widen health inequalities. Conversely, healthy dietary principles can often be preserved through lower-cost substitutions rather than premium or specialist foods. Future trials should report intervention costs and relevant socioeconomic characteristics, and clinical counselling should explicitly account for household resources and food access.

## 4. Defining the Inflammatory Potential of Dietary Patterns

### 4.1. Dietary Inflammatory Index and Related Methodological Considerations

The literature uses terms such as “pro-inflammatory” and “anti-inflammatory” to describe the overall characteristics of dietary patterns rather than the effects of individual foods or nutrients. In this review, these descriptors are considered in the context of the overall dietary composition, including the degree of food processing, fibre and plant-food diversity, fat quality, and the intake of red and processed meat, added sugars and food additives [9,18,19].

The terms “anti-inflammatory diet” and “pro-inflammatory diet” encompass several distinct dietary concepts and do not necessarily denote formal therapeutic interventions. In the literature, the term ‘anti-inflammatory diet’ may refer to named interventions such as the Groningen Anti-Inflammatory Diet (GrAID) or IBD-AID, a broadly plant-forward dietary pattern, or a low score on an inflammatory diet index. Conversely, “pro-inflammatory diet” may refer to an empirically derived Western pattern or to a higher Dietary Inflammatory Index (DII) score. These concepts overlap but are not interchangeable, and neither should be assumed to represent a specific therapeutic protocol.

However, the potential response to a given dietary pattern may also be influenced by the food matrix, the foods used to replace excluded components, the amount and duration of exposure, individual tolerance, disease phenotype and activity, concomitant medications, and nutritional status. Therefore, these terms are used descriptively and should not be interpreted as indicating that a given dietary pattern constitutes a validated anti-inflammatory treatment for IBD [9,18,19].

The DII was developed from a literature-derived scoring algorithm linking dietary parameters to six circulating inflammatory biomarkers: interleukin (IL)-1β, IL-4, IL-6, IL-10, tumour necrosis factor alpha (TNF-α), and C-reactive protein (CRP) [20]. More positive scores indicate a more pro-inflammatory dietary profile, whereas more negative scores indicate a more anti-inflammatory profile. The energy-adjusted DII (E-DII) standardises intake per unit of energy and may reduce confounding by total energy intake. In IBD studies, higher DII scores have been associated with active disease, greater severity, elevated faecal calprotectin, and relapse risk [21,22,23].

However, the DII is not an IBD-specific measure and does not directly quantify mucosal inflammation. Its value depends on the dietary assessment instrument, the number of available food parameters, the accuracy of food-composition databases, and the intake period captured. Food-frequency questionnaires are susceptible to recall bias, while short dietary records may not reflect habitual intake. Studies that calculate the index from different component subsets may not be directly comparable. Furthermore, cross-sectional associations are vulnerable to reverse causation: patients with active symptoms may reduce fibre, fruit, vegetables, dairy products, or fat because of perceived intolerance, thereby producing a more pro-inflammatory score as a consequence rather than a cause of disease activity.

DII-based findings should therefore be interpreted as complementary epidemiological evidence rather than as a prescription for a specific therapeutic diet. Recent work evaluating diet-quality indices in IBD found that available tools differ substantially in the domains captured and that no index has yet been fully validated for an IBD population [18]. Prospective designs repeated dietary measurements, objective inflammatory endpoints, and adjustment for medication, smoking, body composition, socioeconomic status, food security, and physical activity are needed to strengthen causal inference.

### 4.2. Mechanistic Framework and Hierarchy of Clinical Outcomes

Dietary patterns may influence IBD through several interconnected mechanisms. Fermentable fibres and resistant starches provide substrates for microbial production of short-chain fatty acids (SCFAs), including butyrate, which support colonocyte metabolism, epithelial barrier integrity, and immune regulation. Polyphenols and unsaturated fatty acids may also modulate microbial ecology and host signalling. In contrast, low-fibre, high-fat, and highly processed patterns may reduce saccharolytic fermentation, alter bile acid pools, increase mucus degradation, and favour microbial functions associated with endotoxin production [7,8,19,24,25]. These interactions may also involve specific bioactive compounds with immunomodulatory properties. For example, liquiritin apioside was shown in an experimental model to alleviate colitis-associated inflammation by modulating gut microbial metabolites and the Th17/Treg balance [26]. A 2026 observational study further suggests that associations between diet and inflammation may be influenced by distinct microbial features in CD and UC, indicating possible disease-specific biological pathways and generating hypotheses about potential mechanisms that require further investigation [27].

Endoscopic and histological remission provide stronger evidence of disease modification than symptom scores alone. Faecal calprotectin and CRP provide objective information but are imperfect surrogate markers and may not capture all disease locations or phenotypes. Microbiome or metabolomic changes are most informative when linked to reproducible clinical or inflammatory outcomes. Conversely, improvements in dietary quality, nutritional adequacy, or cardiometabolic health may remain worthwhile even when intestinal inflammatory activity is unchanged. The clinical meaning of a dietary intervention therefore depends on the outcome it is intended to achieve.

The impact of diet on the course of IBD should, therefore, be interpreted from multiple perspectives, considering changes in the gut microbiota, objective inflammatory markers, and patient-reported symptoms (Figure 1).

## 5. Minimally Processed, Plant-Forward, and Structured Anti-Inflammatory Patterns

### 5.1. Structured “Anti-Inflammatory” Diets

The GrAID was designed specifically for IBD and combines minimally processed foods with restrictions on processed and preserved foods, sugar-sweetened beverages, and alcohol. Its recommended foods include fish, lean meat, eggs, minimally processed dairy products, vegetables, fruit, legumes, cereals, coffee, tea, and selected natural sweeteners [28]. The IBD-AID similarly emphasises prebiotic and probiotic foods, modification of carbohydrate sources, and staged texture adaptation according to symptoms. These interventions are more structured than general advice to “eat an anti-inflammatory diet”. They are primarily intended to modify inflammatory activity, although their effects on symptoms and nutritional adequacy may also be clinically relevant.

In patients with UC in clinical remission, a six-month anti-inflammatory dietary intervention was associated with favourable metabolomic and microbiome changes and a lower occurrence of subclinical inflammation [29]. A prospective one-year trial also reported a higher likelihood of sustained remission and favourable inflammatory markers among patients who improved dietary quality [30]. Earlier case-series data from Olendzki et al. [31] suggested symptom improvement and reduced medication use, but the uncontrolled design, small sample size, and potential confounding by concurrent treatment limit causal interpretation. A later pilot study supported the feasibility of modulating the microbiome through structured dietary change [24].

Taken together, structured anti-inflammatory diets are promising but remain incompletely standardised. Their apparent effects may reflect several simultaneous changes—including reduced UPF exposure, greater dietary diversity, altered fibre quantity or texture, and intensive dietetic support—rather than a single anti-inflammatory component. Replication in multicentre trials using prespecified objective endpoints is required before any one protocol can be recommended as a universal treatment.

### 5.2. Mediterranean Diet

The MD is not a single rigid prescription but a family of dietary patterns. It is traditionally characterised by high intakes of vegetables, fruits, legumes, nuts, seeds, and minimally processed cereals, with olive oil as the principal culinary fat. It also includes regular fish intake, moderate consumption of dairy products and poultry, and limited consumption of red and processed meat, sweets, and highly processed foods [32,33,34]. Contemporary definitions also recognise social and lifestyle features, although these should be analysed separately from the diet’s nutritional composition.

The MD has several features relevant to IBD, including high fibre and polyphenol density, a favourable unsaturated-to-saturated fat profile, and lower exposure to UPFs. An umbrella review of nutritional exposures found that several dietary factors may be associated with IBD risk or progression. However, the certainty and consistency of the evidence varied across exposures [35]. Greater adherence has been associated with microbial diversity and with taxa and pathways linked to SCFA production [36]. A Mediterranean-like pattern has also been associated with gut microbiome composition and lower subclinical gastrointestinal inflammation, including among individuals with CD [37]. More recent longitudinal data suggest that healthier and Mediterranean-like dietary patterns may relate to inflammatory activity through distinct microbial mediators in CD and UC [27]. These findings strengthen biological coherence but do not by themselves establish treatment efficacy.

Clinical evidence is encouraging but heterogeneous. Earlier systematic reviews reported inverse associations between MD adherence and disease activity or progression [38,39]. A 2026 systematic review and meta-analysis of seven studies involving 223 participants estimated a pooled clinical remission rate of approximately 62% among patients receiving an MD alongside standard medical therapy. However, the MD was not superior to comparison diets for remission induction. Heterogeneity was substantial, and none of the included studies reported endoscopic or histological response. In fact, MD studies are heterogeneous, and the impact of MD on mucosal healing is unknown [39]. Adherence is often lower in patients with stricturing CD, ileocolonic disease, extensive UC, or active symptoms, illustrating the influence of phenotype and reverse causation [40,41]. In mild-to-moderately active UC, interventions incorporating the MD have been associated with improvements in symptoms, quality of life, and selected inflammatory markers. However, interpretation is complicated when dietary and nutraceutical components are combined [42]. Haskey et al. [43] found that a 12-week MD intervention in UC produced favourable changes in faecal calprotectin, secretory immunoglobulin A, and the bacteriome compared with a habitual Canadian diet.

The DINE-CD randomised trial compared the MD with the specific carbohydrate diet (SCD) in adults with CD and mild-to-moderate symptoms. Symptomatic remission at week 6 occurred in 43.5% of participants assigned to the MD and 46.5% assigned to the SCD, with no significant between-group difference. Objective biomarker responses were less frequent than symptomatic responses, particularly for CRP [44]. This distinction is clinically important: both diets may improve symptoms, but the trial did not demonstrate that the more restrictive SCD was superior to the MD for controlling inflammation. The MD is preferred as a broadly applicable starting point because it is less restrictive and offers established cardiovascular and metabolic benefits.

The American Gastroenterological Association advises most patients with IBD to follow a Mediterranean-style pattern rich in diverse plant foods, monounsaturated fats, complex carbohydrates, and lean protein, and low in UPFs, added sugars, and excess salt for overall health and well-being. The same guidance emphasises that no diet has consistently reduced flare rates in all adults with IBD [45]. The 2025 ECCO consensus similarly supports healthy dietary patterns while reserving stronger therapeutic recommendations for selected structured interventions and clinical contexts [9]. Thus, the MD is best framed as a flexible general dietary foundation rather than a stand-alone remission-induction therapy.

The feasibility of an MD also depends on affordability and food access. In some populations, greater adherence has been associated with modestly higher dietary expenditure, and the relative burden may be greater in lower-income households [46,47]. Although economic evaluations suggest that Mediterranean-style eating can be cost-effective at the population level, cost-effectiveness does not guarantee affordability for an individual patient [48]. In IBD, food insecurity may additionally reduce access to tolerated nutrient-dense foods and increase reliance on convenient UPFs [14,49]. The MD should therefore be presented as adaptable principles rather than a list of premium or imported foods; seasonal or frozen produce, staple cereals, legumes when tolerated, eggs, canned fish, and other lower-cost sources of unsaturated fat can preserve its overall direction. Table 1 summarises practical adaptations of Mediterranean dietary principles across stable remission, active symptoms, and clinically significant stricturing phenotypes, based on current ECCO and AGA guidance [9,45].

Dietary recommendations should be individualised according to symptoms, disease activity, and disease phenotype, with particular attention to food form and consistency, as well as fibre content, especially during active disease and in patients with stricturing disease.

The practical objective is not to reproduce every element of a traditional Mediterranean pattern during every phase of disease. Rather, the aim is to preserve its overall direction—greater dietary diversity when feasible, a preference for minimally processed foods and unsaturated fats, and lower exposure to processed meat and UPFs—while adapting texture, fibre load, energy density, and food choice to disease phenotype and tolerance. Texture modification should not be equated with permanent fibre avoidance. IBD-specialist dietetic input is particularly important in patients with strictures, short bowel, high-output stoma, active weight loss, or multiple self-imposed exclusions [9,45].

### 5.3. Plant-Forward, Vegetarian, and Semi-Vegetarian Patterns

Plant-forward dietary patterns increase the contribution of vegetables, fruit, legumes, cereals, nuts, seeds, and plant-derived oils without necessarily eliminating all animal foods. This broader category is more clinically useful than treating vegetarianism as a homogeneous exposure. Lacto-ovo vegetarian, pesco-vegetarian, vegan, and semi-vegetarian diets differ substantially in protein sources, fibre content, micronutrient density, and degree of processing [50].

Vegetarian diets can provide vitamin C, vitamin E, polyphenols, dietary fibre, and unsaturated fatty acids, but they are not inherently anti-inflammatory. A pattern dominated by refined grains, confectionery, sugar-sweetened beverages, meat substitutes, and other UPFs may have poor nutritional quality, even when excluding meat [50]. In general populations, vegetarian diets have been associated with lower energy-adjusted DII scores in some cohorts, while meta-analyses suggest lower CRP concentrations; findings for individual cytokines, including IL-6, remain inconsistent [36,50,51]. Such evidence cannot be directly extrapolated to patients with active IBD.

Evidence in established IBD is limited. Jowett et al. [52] reported an association between higher meat intake, particularly red and processed meat, and UC relapse. In a small, non-randomised study, a semi-vegetarian diet permitting fish weekly and meat every two weeks was associated with prolonged remission in CD [53]. The reported effect was substantial, but the study was conducted in a specific clinical setting and included intensive lifestyle support; replication in larger randomised studies is needed. The ECCO consensus therefore identifies vegetarian strategies as an area of interest with insufficient evidence for a firm recommendation [9].

The feasibility of a plant-forward pattern depends on disease activity and phenotype. Legumes and large quantities of intact fibre may exacerbate bloating, pain, or stool frequency without necessarily increasing inflammation. In active disease, stricturing CD, or patients with high nutritional requirements, exclusive reliance on plant proteins can make adequate protein and energy intake more difficult. Vegan diets also require particular attention to vitamin B12, iron, zinc, calcium, iodine, vitamin D, and n-3 fatty acids [54]. A pragmatic recommendation is therefore to increase tolerated minimally processed plant foods and reduce processed meat while preserving sufficient protein and micronutrient intake, rather than promoting universal meat exclusion.

## 6. Western and Ultra-Processed Food-Rich Dietary Patterns

### 6.1. Association with IBD Development

The Western diet (WD) is the best-characterised dietary pattern with pro-inflammatory features. It commonly combines a high energy density and glycaemic load with high intakes of refined grains, added sugars, red and processed meat, animal fat, high-fat dairy products, fast foods, and UPFs and low intakes of vegetables, fruit, whole grains, and fibre [55,56]. Because these components cluster together, it is difficult to isolate the contribution of any single nutrient.

Prospective and case–control studies associate Western or meat-rich dietary patterns with a greater likelihood of developing IBD. In the large population-based cohort reported by Peters et al. [55], Western and empirically derived “carnivorous” patterns were associated with incident IBD. Importantly, the latter was a statistical dietary pattern identified within a cohort and should not be treated as direct evidence about an intentional all-meat carnivore diet. Higher early-life intake of processed and ultra-processed foods has also been reported among patients with CD compared with control groups [57]. Earlier Japanese data linked a pattern including bread, butter, and sausage to UC risk [58].

The strongest prospective evidence concerns UPFs. In the PURE cohort, higher UPF consumption was associated with incident IBD in a dose-dependent manner; compared with fewer than one serving per day, one to four and at least five servings per day were associated with progressively higher risk [59]. These associations persisted after multivariable adjustment, but residual confounding remains possible because UPF consumption also tracks with socioeconomic and lifestyle factors. Systematic reviews of pre-diagnostic diet likewise suggest that high intakes of animal fat, meat, and refined foods, and low intakes of fruit or fibre may be associated with IBD. However, older studies are heterogeneous and often retrospective [60].

### 6.2. Disease Activity, Relapse, and Dietary Inflammatory Potential

In established IBD, cross-sectional studies generally report more pro-inflammatory DII scores in active disease than in remission. However, these differences should be interpreted with caution, as dietary changes may also result from symptoms or disease activity rather than cause them. Patients experiencing gastrointestinal symptoms may restrict fibre-rich foods, fresh fruits and vegetables, dairy products, or other foods perceived as symptom triggers. Such restrictions may reduce dietary diversity and alter the relative contribution of processed foods to the overall diet. Therefore, reverse causality and symptom-driven dietary changes may influence associations between dietary patterns and disease activity [10,61].

Bakhtiari et al. [22] found that patients with UC consuming more pro-inflammatory diets had greater disease severity. Rocha et al. [23] observed that the highest DII quartile was associated with higher faecal calprotectin, zonulin, and a greater abundance of Veillonella rogosae; these variables predicted relapse over six months.

Evidence concerning red and processed meat differs between UC and CD. A prospective UC cohort found a higher relapse risk among patients with the highest meat intake, particularly red and processed meat [52]. The large multicentre PREdiCCt prospective cohort followed 2629 patients in self-reported remission for a median of 4.1 years and provided more recent, disease-specific evidence. In UC, participants in the highest quartile of total meat intake had a higher risk of objective flare. No consistent associations were observed for UPFs, fibre, or polyunsaturated fatty acids [62]. In contrast, an intervention reducing red and processed meat in CD did not consistently reduce symptomatic relapse. The most defensible interpretation is therefore that habitual meat intake may be relevant to relapse susceptibility in UC. At the same time, evidence remains insufficient for universal meat-exclusion advice across all IBD phenotypes.

### 6.3. Potentially Relevant Components and Mechanisms

Low fibre intake may reduce substrates for SCFA production and encourage microbes to use host-derived glycans. This may weaken mucus-associated ecological functions and alter epithelial and immune homeostasis [19,24,25]. At the same time, fibre is not a single entity: fermentability, solubility, particle size, and the food matrix influence tolerance and microbial effects. The clinical response to an intact legume, a purified supplement, and a finely milled cereal cannot be assumed to be equivalent.

Dietary fat quality may also be relevant. Experimental studies indicate that saturated and oxidised fatty acids can influence innate immune signalling, epithelial permeability, and microbial composition [6,8]. Long-chain fatty acids have been shown to promote colitis in mechanistic models through pathways involving CD36-mediated uptake and STAT3 palmitoylation [63]. These data do not establish a direct clinical effect of a specific fat in humans, but they support examining fat type rather than total fat intake alone. Diets combining high saturated fat with refined sugars may be particularly unfavourable; in mice, a high-fat diet with a concentrated sucrose solution aggravated mucosal inflammation and altered the microbiome [64].

Meat may also influence the intestinal environment through mechanisms related to its specific components, particularly haem iron. Haem iron, found in higher amounts in red meat, may act as a pro-oxidant factor and promote lipid peroxidation, with subsequent formation of reactive aldehydes and other potentially harmful compounds during digestion. These processes may compromise epithelial integrity and alter the intestinal microbial environment [65]. Such mechanisms provide biological plausibility for potential associations between red or processed meat intake and the occurrence or course of IBD. Still, they do not establish a causal relationship between meat consumption and IBD outcomes in humans [19].

Food additives are an important hypothesis linking industrial diets with IBD. Experimental studies indicate that emulsifiers, such as polysorbate 80 and carboxymethylcellulose, and thickeners, such as carrageenan, may alter the microbial composition, interactions with the mucus layer, and inflammatory responses [7,66,67]. A previous small randomised study associated carrageenan supplementation with more frequent UC relapses, although the sample size was limited and the exposure may not reflect typical dietary intake [68]. A short-term intervention with a low-emulsifier diet in CD was feasible and improved food-related quality of life and symptoms, but did not show sustained endoscopic efficacy [69]. Importantly, a double-anonymised, placebo-controlled study in healthy adults in 2026 showed that supplementation with five commonly used emulsifiers did not increase faecal calprotectin or CRP concentrations over four weeks. However, several emulsifiers were associated with lower SCFA concentrations, and carrageenan increased intestinal permeability [70]. However, these findings should not be directly extrapolated to patients with IBD, particularly those with active disease, given the short intervention period, the limited number of emulsifiers studied, and the inclusion of healthy adults. Overall, human studies indicate biological effects of selected food additives, but the available evidence remains insufficient to conclude that usual exposure to emulsifiers directly causes or exacerbates IBD.

Other additives, including titanium dioxide, non-nutritive sweeteners, and preservatives, have been implicated mainly through experimental or observational evidence [56,71,72,73]. Exposure is difficult to quantify, and UPFs vary widely in nutrient profile, food matrix, additives, cost, and convenience. It is therefore inappropriate to treat every UPF or additive as biologically equivalent. A pragmatic clinical message is to reduce habitual reliance on heavily processed foods where feasible and to favour nutritionally adequate, minimally processed alternatives, rather than providing long lists of individual additives that patients should avoid without direct clinical evidence.

High salt intake may promote T-helper-17-related immune responses in experimental systems, while excess added sugar may contribute to an unfavourable dietary pattern and microbial metabolism [71,74]. These findings support moderation, but they do not justify aggressive sodium restriction in patients with high-output diarrhoea or a stoma, in whom fluid and electrolyte replacement may be necessary. Clinical recommendations must therefore integrate inflammatory hypotheses with the patient’s immediate physiological needs.

FODMAPs require separate consideration. Poorly absorbed fermentable carbohydrates can increase luminal water and gas, thereby provoking bloating, pain, and altered stool frequency [75]. These symptom-generating mechanisms do not establish a pro-inflammatory effect. Conflating FODMAP intolerance with mucosal inflammation may lead patients to interpret symptom relief as disease control or to unnecessarily continue restrictive diets.

## 7. Popular Restrictive Dietary Patterns with Insufficient Evidence in IBD

### 7.1. Ketogenic Diet

A ketogenic diet (KD) is characterised by severe carbohydrate restriction and sufficient fat intake to induce nutritional ketosis. Its composition varies considerably; some versions rely heavily on meat, dairy fat, coconut oil, and processed low-carbohydrate products, whereas others use olive oil, nuts, seeds, avocado, fish, and low-carbohydrate vegetables. A KD should therefore not be automatically equated with a carnivore diet or high saturated fat intake.

Ketone bodies can modulate cellular metabolism and selected inflammatory pathways, creating a theoretical rationale for benefit. Conversely, severe carbohydrate restriction may reduce fibre, resistant starch, fruits, legumes, and whole grains, potentially lowering microbial substrate diversity. The net effect likely depends on food quality, fat composition, duration, and the baseline microbiome. The available reviews of ketogenic diets are insufficient to establish their safety or efficacy in IBD [76].

Direct human evidence for the KD in IBD remains inadequate. Associations between the overall inflammatory potential of habitual diet and either disease activity [21] or incident IBD [77] do not test a ketogenic intervention, and mechanistic studies of long-chain fatty acids cannot be generalised to every ketogenic formulation [63,78]. Potential concerns include reduced fibre and micronutrient intake, hyperlipidaemia, constipation, loss of dietary diversity, and difficulty maintaining adequate energy intake during active disease [76,78]. Accordingly, a KD cannot currently be recommended as routine anti-inflammatory therapy for IBD; future trials would need to define diet composition precisely and distinguish well-formulated ketogenic diets from high-saturated-fat or carnivore patterns.

### 7.2. Intentional Carnivore Diet

The intentional carnivore diet consists almost exclusively of meat, eggs, animal fats, and sometimes dairy products, with near-complete exclusion of plant foods. This should be distinguished from a predominantly animal-based diet resulting from restricting or eliminating plant foods such as vegetables, fruits, legumes, or other foods perceived to worsen gastrointestinal symptoms. In such cases, dietary restriction may be driven by symptom management rather than by the intentional adoption of a carnivore diet. It differs conceptually from a high-meat dietary pattern identified through epidemiological analysis. This dietary pattern provides virtually no dietary fibre. It eliminates most plant-derived polyphenols, while potentially providing high amounts of saturated fat, haem iron and processed meat depending on food choices. These features raise concerns about microbial diversity, bowel function, cardiometabolic risk, and micronutrient adequacy [19,79].

Animal-protein-rich diets have been associated with microbial profiles enriched in taxa and pathways linked to endotoxin production, and an animal-protein-rich diet exacerbated experimental colitis compared with a plant-protein-rich diet [25,80]. These observations support caution but do not constitute direct evidence that an intentional carnivore diet worsens IBD in humans. Conversely, a case series of 10 self-selected patients reported improved IBD-related quality of life while following a carnivore-ketogenic diet [81]. The absence of a control group, reliance on self-report, heterogeneous disease status, and lack of systematic endoscopic assessment make the results highly uncertain.

The available evidence is insufficient to classify the intentional carnivore diet as either a proven pro-inflammatory exposure or an effective treatment. Its extreme restriction, absence of prospective controlled efficacy data, and uncertain long-term nutritional and cardiometabolic safety argue against routine use. Clinicians should address patient interest non-judgementally, emphasising nutritional assessment, objective disease monitoring, and the risk of substituting an unproven dietary approach for effective medical therapy.

Ketogenic and carnivore diets may also have adverse effects on the lipid profile, particularly when these diets are high in saturated fat. This may be clinically relevant in patients receiving corticosteroids, which can independently contribute to adverse metabolic and cardiovascular risk profiles.

The reasons patients choose ketogenic or carnivore diets are likely multifactorial. Some patients encounter claims in non-scientific or non-professional sources suggesting that these diets have anti-inflammatory effects. Others may experience subjective symptom improvement after reducing fermentable or fibre-rich foods, although this should not be interpreted as evidence of reduced intestinal inflammation. Interest in restrictive diets may also arise when patients perceive conventional treatment as insufficient and seek alternative approaches.

## 8. Low-FODMAP Diet for Persistent Functional Symptoms

A low-FODMAP diet targets different symptoms. It is intended to reduce fermentative and osmotic symptoms in patients with irritable bowel syndrome or IBS-like symptoms, including patients with quiescent IBD. In a randomised trial of patients with quiescent IBD and persistent gastrointestinal symptoms, low-FODMAP advice improved selected symptoms and provided adequate relief but did not significantly change inflammatory markers; it also reduced the abundance of several bacterial species generally considered beneficial [82]. The diet should therefore not be presented as treatment for active intestinal inflammation.

When used, the restrictive phase should be time-limited and followed by structured reintroduction and personalisation, ideally under dietetic supervision. Before attributing symptoms to functional mechanisms, clinicians should evaluate for active inflammation, infection, bile acid diarrhoea, small-intestinal bacterial overgrowth, strictures, and other treatable causes [9,45]. Eliminate as few products as possible. The aim should be the most varied diet possible with the fewest eliminations possible.

## 9. Translation to Clinical Practice

### 9.1. Match the Dietary Strategy to the Clinical Objective

The choice of dietary strategy should be guided by the intended clinical objective, while considering disease activity, symptom burden, nutritional status, and the patient’s reasons for adopting dietary modification. For general health, cardiometabolic risk reduction, and improvement in overall diet quality, a cost-adapted Mediterranean-style pattern is a reasonable default. During active symptoms, short-term modification of texture, particle size, fibre load, or fat may improve tolerance but should not replace assessment and treatment of active inflammation. In objectively quiescent IBD with persistent IBS-like symptoms, a time-limited low-FODMAP intervention may be appropriate. Assess disease activity and symptom burden separately.

The safety and potential benefits of specific dietary patterns among patients with IBD depend on the diet’s nature, including the presence or restriction of animal-derived foods, its restrictiveness, the content of individual nutrients, and disease activity and individual tolerance. Available evidence suggests that restrictive diets may increase the risk of nutritional deficiencies. Therefore, such diets should be introduced cautiously, taking into account the patient’s individual needs.

Table 2 compares the principal clinical roles, available evidence, current clinical positions, and major limitations of the popular dietary patterns discussed in this review.

### 9.2. Individualise According to Phenotype and Nutritional Risk

Dietary tolerance varies according to disease location, strictures, previous surgery, bile acid malabsorption, pancreatic insufficiency, stoma output, and concurrent functional disorders. In clinically significant stricturing CD, modification of fibre texture and particle size may be required, whereas indiscriminate long-term fibre restriction in non-stricturing disease may reduce dietary diversity. Patients with extensive small-bowel disease, active weight loss, sarcopenia, or high-output stoma may prioritise energy, protein, fluid, and electrolyte requirements over idealised healthy-eating targets.

Nutritional assessment should include weight trajectory, dietary intake, micronutrient risk, body composition (when available), and the number and rationale for excluded foods. Iron, vitamin B12, folate, vitamin D, calcium, zinc, and magnesium may require targeted evaluation according to phenotype and treatment. Dietary interventions are more likely to be sustainable when they involve graded changes, culturally appropriate foods, and shared decision-making rather than long lists of prohibitions.

Involving a dietitian with IBD expertise is particularly valuable when implementing EEN, CDED, a low-FODMAP diet, vegan diets, extensive elimination diets, or dietary modification in stricturing disease. Professional support can also reduce the risk of conflating symptom triggers with inflammatory triggers and can facilitate the reintroduction of unnecessarily excluded foods [9,45].

### 9.3. Affordability, Food Security, and Equitable Implementation

A dietary recommendation is clinically useful only if the patient can obtain, prepare, tolerate, and sustain it. Nutritional assessment should therefore extend beyond weight loss and laboratory deficiencies to include food insecurity, competing household expenses, refrigeration and cooking facilities, work schedules, disability, and local availability of suitable foods. In 2023 US survey data, food insecurity was reported by 13.48% of adults with IBD compared with 8.96% of those without IBD, and IBD remained independently associated with food insecurity after adjustment [14]. Although these data are not directly generalisable to every healthcare system, they show that economic barriers affect dietary care implementation.

Equitable implementation requires flexible, rather than premium, versions of dietary patterns. Dietitians should identify lower-cost substitutions, avoid unnecessary specialist products, and prioritise the clinical objective most relevant to the individual patient. During active weight loss, high-output diarrhoea, or severe nutritional compromise, adequate energy, protein, fluid, and electrolyte intake may take precedence over strict adherence to an idealised healthy-eating pattern. Research protocols should report intervention cost, participant socioeconomic characteristics, reimbursement or food provision, and the time and equipment required for shopping and preparation, because these variables directly affect adherence and external validity [10,14,46,47,48,49].

In general, dietary modifications should be considered on an individual basis, as the same dietary pattern may have different implications depending on disease activity, symptoms, tolerance, and the patient’s nutritional and socioeconomic circumstances.

Table 3 summarises how these patient-accessible patterns are typically used during remission and during active symptoms, along with their main accessibility, equity, and nutritional risks [9,10,45,49,61,82].

## 10. Discussion

Dietary evidence in IBD is best interpreted across distinct clinical domains rather than along a single “anti-inflammatory–pro-inflammatory” axis. A major contribution of this review is to present this complexity from a patient-centred perspective that considers real-world circumstances. Patients select or eliminate specific foods not only based on evidence related to inflammation but also on symptoms, concerns about disease relapse, cost, convenience, cultural factors, available online information, and access to professional support [10,11]. Recent reviews have comprehensively summarised protocolised dietary therapies and their effects on the gut microbiome [12]. In contrast, the present synthesis focuses on everyday dietary choices that patients may make outside research protocols. These choices are evaluated in terms of objective inflammation, symptom control, nutritional adequacy and safety, disease phenotype, and accessibility.

Among general dietary patterns, the Mediterranean diet (MD) has one of the most extensively studied profiles in terms of nutritional value, number of clinical studies, feasibility, and health benefits demonstrated outside IBD [32,33,34,36,37,38,39,84]. Nevertheless, a 2026 meta-analysis highlights the need for cautious interpretation: clinical remission was frequently observed, but MD did not demonstrate superiority over comparator diets, and objective mucosal outcomes were not available [39]. The DINE-CD trial provides similar information, as symptomatic remission was frequently achieved with both MD and SCD, whereas responses based on objective biomarkers were less common [44]. This discrepancy between symptoms and inflammatory activity is crucial for interpreting dietary studies. It supports presenting MD as a flexible foundation for a healthy dietary pattern rather than a proven treatment that leads to mucosal healing.

Plant-forward and semi-vegetarian diets represent potential alternatives to, or variations of, MD; however, the evidence base for their use in IBD is more limited [53,54]. A potentially important component of such dietary patterns may be replacing processed meat and refined products with a variety of minimally processed plant foods, rather than completely excluding all animal-derived foods. This distinction is clinically relevant because highly restrictive vegan diets may increase the risk of nutritional deficiencies in patients with active inflammation or malabsorption [5,50,54]. In contrast, well-planned, flexible plant-predominant dietary patterns may support adequate protein and energy intake [54].

Western dietary patterns and higher consumption of ultra-processed foods (UPF) show the most consistent unfavourable epidemiological associations, particularly with the risk of developing IBD [55,59,60,77]. The PURE study indicates an association between higher UPF consumption and disease risk [59], while microbiome studies provide mechanistically consistent observations [25,81]. At the same time, the PREdiCCt study did not demonstrate consistent associations between UPF consumption and objectively assessed disease exacerbation in patients with established IBD. In contrast, higher total meat intake was associated with objectively assessed disease exacerbation specifically in UC [62]. Experimental data concerning individual food additives are also more complex than the findings from preclinical studies. In a recent placebo-controlled randomised trial in healthy individuals, four-week supplementation with five food emulsifiers altered selected microbial metabolites and measures of intestinal permeability. Still, it did not increase faecal calprotectin or CRP concentrations [70]. These findings illustrate why evidence concerning disease development, mechanisms, and relapse should not be combined into a single causal pathway.

The review also highlights the risk of overinterpreting findings related to emerging restrictive dietary patterns. A “carnivorous” dietary pattern identified from dietary data in a cohort study should not be equated with an intentionally adopted carnivore diet as a defined dietary pattern, because these represent different types of dietary exposure [55,81]. Associations between saturated fat intake and IBD do not allow conclusions about the effects of every ketogenic diet [8,76]. Similarly, uncontrolled reports of improved health outcomes cannot confirm efficacy [81]. Therefore, it is more scientifically appropriate to present ketogenic and carnivore diets as dietary patterns with uncertain effects rather than to classify them categorically as either pro-inflammatory or therapeutic.

Protocolised dietary therapies specifically developed for patients with IBD should be distinguished from popular dietary patterns. Interventions such as exclusive enteral nutrition (EEN), CDED combined with PEN, and CD-TREAT were developed and evaluated within defined therapeutic protocols; therefore, their evidence base differs from that of the dietary patterns discussed in the present review [9,44,49,61].

Several limitations affect the interpretation of the current evidence. Definitions of dietary patterns are inconsistent, dietary interventions are difficult to blind, adherence is often assessed by self-report, and control conditions may include either habitual diet or another active intervention. Changes in pharmacological treatment may act as confounding factors, while patients with severe active disease are often underrepresented. Many studies combine patients with CD and UC, include individuals with or without objective evidence of inflammation, or use short follow-up periods. Microbiome findings depend on sequencing methods, geography, baseline dietary patterns, and treatment, whereas dietary quality indices have rarely been validated specifically in IBD [18]. Differences in disease phenotype and location, disease activity, pharmacological treatment, nutritional status, duration of dietary exposure, adherence, and definitions of individual dietary patterns further complicate interpretation of popular dietary patterns [12,18]. Most interventional studies also insufficiently account for food costs, access to food products, socioeconomic status, the burden associated with food preparation, or household food security, limiting the generalisability of the findings to real-world settings [14,46,47,49]. Accordingly, the current evidence should be interpreted with appropriate caution.

This review also has methodological limitations. It is a structured narrative review rather than a systematic review; we selected studies thematically and did not apply a formal tool to assess risk of bias or certainty of evidence. The broad scope of the research question also required the inclusion of heterogeneous study designs, ranging from randomised trials to observational, qualitative, and mechanistic studies. This approach allows the integration of clinical effectiveness with patient behaviours and intervention feasibility but also increases susceptibility to selection and interpretive bias. The conclusions should therefore be regarded as a clinically oriented synthesis of the current evidence rather than therapeutic recommendations supported by a defined level of certainty.

## 11. Future Research and Implementation Challenges

The topic of diet in inflammatory bowel disease should be continually expanded and further explored. Future studies should assess not only gastrointestinal symptoms but also objective inflammatory outcomes, including endoscopic activity and relevant inflammatory biomarkers.

One of the major challenges is selecting sufficiently homogeneous patient populations. Pharmacological therapies may modify the effects of dietary interventions and should therefore be considered in future study designs. Future research should also investigate whether nutritional therapy can be tailored to concomitant pharmacological treatments. Conversely, the selection of pharmacotherapy might eventually take dietary preferences and nutritional deficiencies into account.

On the other hand, dietary studies appear to be particularly challenging because they often require substantial changes in patients’ lifestyles. It should be remembered that patients with inflammatory bowel disease frequently eliminate various foods from their diets due to fear of symptom exacerbation and food aversions.

Additionally, an interesting area of research would be to investigate how diet shapes the gut microbiome and to design studies assessing the effects of specific dietary patterns. It would also be valuable to determine whether gut microbiome composition influences patients’ dietary preferences.

Such studies should also consider the potential adverse effects of the dietary interventions employed. For example, a ketogenic diet may adversely affect the lipid profile, which may already be impaired as a result of corticosteroid therapy.

## 12. Conclusions

The main conclusions of this review are as follows:

1. Current evidence supports a flexible, cost-adapted Mediterranean-style pattern as the best-supported general dietary framework for many patients with IBD, but not as a universally proven therapy for mucosal healing.
2. A pragmatic approach is to increase the diversity of tolerated minimally processed plant foods, favour unsaturated fats, ensure adequate and affordable protein, and reduce habitual reliance on processed meat, excess added sugar, and UPFs.
3. The amount, texture, and particle size of fibre should be adapted to disease activity, stricturing phenotype, and individual tolerance rather than restricted routinely.
4. Western and UPF-rich patterns are consistently associated with less favourable epidemiological data for IBD development. However, their relationship with flares in established disease is less uniform, and evidence for individual additives remains incomplete.
5. Low-FODMAP approaches may improve selected symptoms, but symptom benefit should not be interpreted as evidence of inflammatory control, and prolonged exclusions require reassessment.
6. Evidence for ketogenic and intentional carnivore diets remains insufficient to support their routine use. Across all dietary strategies, affordability, food security, cooking resources, phenotype, nutritional risk, and access to specialist dietetic care should be treated as core clinical variables.
7. Dietary management should complement, not replace, established medical therapy and should aim to expand, rather than unnecessarily narrow, the patient’s diet whenever disease phenotype and tolerance permit.

## Figures and Tables

**Figure 1:**
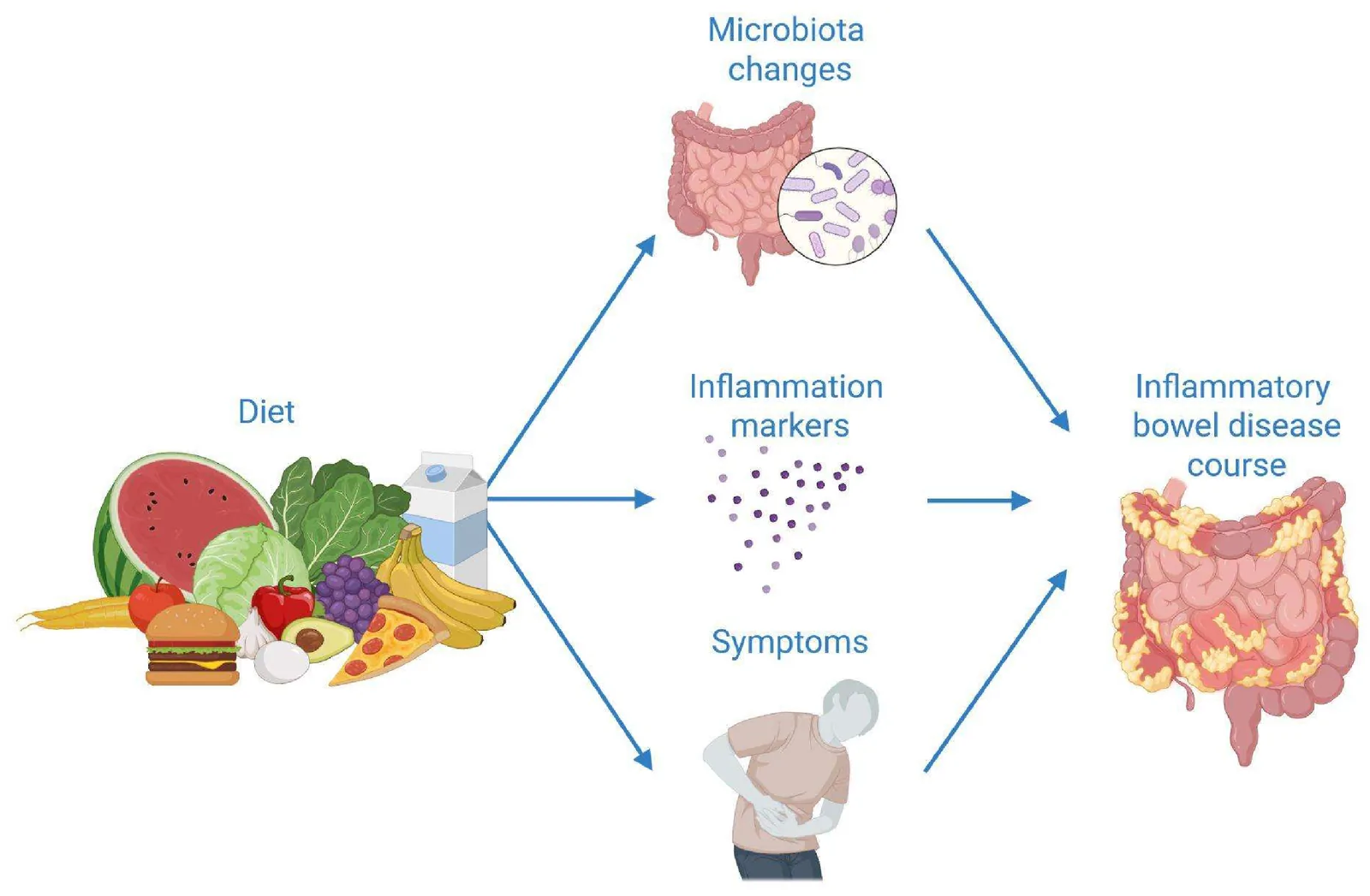
Conceptual framework illustrating the multidimensional relationship between diet and the course of inflammatory bowel disease (IBD), including potential effects on the gut microbiota, inflammatory markers, and gastrointestinal symptoms. Created with BioRender.com.

**Table 1:** Practical adaptation of Mediterranean dietary principles according to IBD activity and phenotype.

Mediterranean Component	During Stable Remission	During Active Symptoms and/or Clinically Significant Strictures	Clinical Caveat
Vegetables and fruit	Aim for variety and gradual expansion of tolerated plant foods.	Modify texture and particle size by peeling, cooking, blending, or sieving; prioritise tolerated options.	Restriction should be symptom- and phenotype-driven rather than automatic.
Whole grains and fibre-rich cereals	Use whole grains when tolerated and when no obstructive phenotype is present.	Temporarily use lower-residue or refined products if clinically indicated.	Targeted fibre restriction is most relevant to symptomatic strictures; prolonged restriction may reduce dietary diversity.
Legumes	Introduce gradually; use smaller portions and well-cooked or blended forms.	Reduce or avoid temporarily when they clearly worsen symptoms; tofu or smooth spreads may be easier to tolerate.	Symptoms related to fermentation do not necessarily indicate increased inflammation.
Nuts and seeds	Use whole, ground, or butter forms according to tolerance.	Prefer smooth nut butters or finely ground preparations; avoid large particles in clinically significant strictures.	Account for energy density in undernutrition and obesity.
Olive oil and other unsaturated fats	Use olive oil as the principal culinary fat.	Continue if tolerated; adjust total fat when steatorrhoea, bile acid malabsorption, or severe diarrhoea is present.	Fat tolerance varies after ileal disease or resection.
Fish, poultry, dairy, and eggs	Include as protein sources according to preference and nutritional requirements.	Choose easily tolerated forms; use lactose-free dairy when lactose malabsorption is present.	A blanket dairy exclusion is not supported unless intolerance or allergy is documented.
Red and processed meat	Limit processed meat and keep red meat intake moderate.	Avoid using processed meat as a convenient substitute for poorly tolerated plant foods.	Evidence linking meat intake to relapse is more consistent in UC than in CD; routine complete meat exclusion is not established.
Hydration and electrolytes	Maintain adequate fluid intake.	Individualise fluid and sodium replacement in high-output diarrhoea or stoma.	Routine salt restriction may be inappropriate when gastrointestinal sodium losses are substantial.
Affordability and food access	Use seasonal or frozen produce, staple cereals, legumes, eggs, and canned fish according to tolerance and preference.	Prioritise tolerated, energy-dense, low-cost options; adapt preparation methods rather than relying on specialist products.	Screen for food insecurity, household resources, and access to cooking facilities. Expensive imported or premium foods are not essential to the pattern.

**Table 2:** Comparative interpretation of major dietary patterns and nutritional therapies in IBD.

Pattern/Intervention	Primary Clinical Role	Evidence Summary	Current Clinical Position	Major Limitations
Mediterranean diet	General dietary framework; overall health; possible adjunct in maintenance	Observational and interventional data suggest benefits for diet quality, symptoms, microbiome features, and selected biomarkers. A 2026 meta-analysis found no superiority over control diets for clinical remission and no endoscopic or histological outcome data [35,39,44,45].	Reasonable cost-adapted general framework; adapt texture and fibre to phenotype and symptoms. Not a stand-alone remission-induction therapy.	Variable definitions and outcomes; affordability and food access may limit adherence.
Plant-forward/ semi-vegetarian	Improve diet quality and reduce processed meat exposure	Potentially protective associations; limited IBD-specific evidence, with one small semi-vegetarian study suggesting prolonged CD remission [35,50,53,54,83]	May be considered when nutritionally adequate and tolerated.	Sparse RCT data; risk of protein and micronutrient inadequacy with restrictive versions.
Western/ UPF-rich pattern.	Exposure/ risk pattern rather than therapy.	Higher intake is consistently associated with incident IBD, especially for UPFs; associations with flare in established IBD are less consistent [55,59,62].	Reduce habitual UPF and processed meat intake.	Residual confounding; heterogeneous UPFs; limited intervention data.
Ketogenic diet	Experimental/ Restrictive pattern	Insufficient direct human evidence; mechanistic findings are mixed and depend on diet formulation [63,76,77,78].	Not recommended routinely for IBD treatment.	Highly variable formulation; low fibre; nutritional and cardiometabolic concerns.
Carnivore diet	Experimental/ restrictive pattern.	No direct prospective evidence for the intentional diet; only uncontrolled case-level reports in established IBD [19,79,81].	Not recommended outside research settings.	Extreme restriction; no long-term safety or objective efficacy data.
Low-FODMAP diet	Persistent functional symptoms in quiescent IBD	Can improve selected symptoms but does not consistently reduce objective inflammation [82].	Short-term, dietitian-guided use with reintroduction.	Restrictive; may reduce beneficial taxa and dietary diversity.

**Table 3:** Real-world use of patient-accessible dietary patterns according to disease activity, accessibility, and principal risk.

Pattern	Typical Use in Remission	Typical Use During Active Symptoms	Accessibility, Equity, and Principal Risk
Mediterranean	General diet-quality framework; may support symptoms, general health, and maintenance.	Retain core direction but modify texture, fibre load, fat, and portion size according to phenotype and tolerance.	Can be affordable when based on staple foods, but premium interpretations may be inaccessible; screen for food insecurity.
Plant-forward/ vegetarian	May increase dietary diversity and reduce processed meat when nutritionally adequate.	Intact legumes and high fibre may be poorly tolerated; energy and protein adequacy become priorities.	Low-cost versions are possible, but specialist meat substitutes may increase cost and UPF exposure.
Western/UPF-rich	Often reflects habitual convenience eating rather than a therapeutic choice.	May become more prominent when tolerated packaged foods replace fresh foods during symptoms.	Highly accessible and convenient; low price, shelf stability, and low preparation burden may reinforce use when food security is limited.
Low-FODMAP	May improve persistent IBS-like symptoms in objectively quiescent IBD.	Not a treatment for active inflammation and may be unnecessarily restrictive during flare.	Requires education and reintroduction; specialist products and dietetic input may increase cost.
Ketogenic/ carnivore	Popular self-directed restrictive options with insufficient objective IBD evidence.	High fat and severe restriction may be poorly tolerated or nutritionally inappropriate.	Can be expensive, socially restrictive, and difficult to balance; long-term safety is uncertain.

Note: Typical use reflects reported or clinically encountered behaviour and does not imply population prevalence, anti-inflammatory efficacy, or suitability for all patients. Assess disease activity objectively when using dietary changes to guide management.

## Data Availability

No new data were created or analysed in this study. Data sharing is not applicable to this article.

## Abbreviations

CD, Crohn’s disease; CDED, Crohn’s Disease Exclusion Diet; CRP, C-reactive protein; DII, Dietary Inflammatory Index; DINE-CD, Diet to INducE Remission in Crohn’s Disease; E-DII, energy-adjusted Dietary Inflammatory Index; EEN, exclusive enteral nutrition; FODMAPs, fermentable oligosaccharides, disaccharides, monosaccharides, and polyols; GrAID, Groningen Anti-Inflammatory Diet; IBD, inflammatory bowel disease; IBD-AID, Inflammatory Bowel Disease Anti-Inflammatory Diet; KD, ketogenic diet; MD, Mediterranean diet; PEN, partial enteral nutrition; PREdiCCt, PRognostic effect of Environmental factors in Crohn’s and Colitis; PURE, Prospective Urban Rural Epidemiology; SCD, specific carbohydrate diet; SCFAs, short-chain fatty acids; UC, ulcerative colitis; UPFs, ultra-processed foods; WD, Western diet.

## References

[1] Gisbert J.P., Chaparro M., Verstockt B., Kienle P., Minozzi S., Allocca M., Kucharzik T. (2026). ECCO Guidelines on Therapeutics in Ulcerative Colitis: Medical Treatment. J. Crohn’s Colitis.

[2] Gordon H., Minozzi S., Kopylov U., Verstockt B., Chaparro M., Buskens C., Raine T. (2024). ECCO Guidelines on Therapeutics in Crohn’s Disease: Medical Treatment. J. Crohn’s Colitis.

[3] Yanai H., Ellul P., Verstockt B., Greuter T., Kopylov U., Casanova M.J., Gisbert J.P. (2026). ECCO Guidelines on the Prevention, Diagnosis, and Management of Infections in Inflammatory Bowel Disease. J. Crohn’s Colitis.

[4] van Linschoten R.C.A., Visser E., Niehot C.D., van der Woude C.J., Hazelzet J.A., van Noord D., West R.L. (2021). Systematic review: Societal cost of illness of inflammatory bowel disease is increasing due to biologics and varies between continents. Aliment. Pharmacol. Ther..

[5] Rizzello F., Gionchetti P., Spisni E., Saracino I.M., Bellocchio I., Spigarelli R., Collini N., Imbesi V., Dervieux T., Alvisi P. (2023). Dietary Habits and Nutrient Deficiencies in a Cohort of European Crohn’s Disease Adult Patients. Int. J. Mol. Sci..

[6] Keewan E., Narasimhulu C.A., Rohr M., Hamid S., Parthasarathy S. (2020). Are Fried Foods Unhealthy? The Dietary Peroxidized Fatty Acid, 13-HPODE, Induces Intestinal Inflammation In Vitro and In Vivo. Antioxidants.

[7] Bancil A.S., Sandall A.M., Rossi M., Chassaing B., Lindsay J.O., Whelan K. (2021). Food Additive Emulsifiers and Their Impact on Gut Microbiome, Permeability, and Inflammation: Mechanistic Insights in Inflammatory Bowel Disease. J. Crohn’s Colitis.

[8] Basson A.R., Chen C., Sagl F., Trotter A., Bederman I., Gomez-Nguyen A., Sundrud M.S., Ilic S., Cominelli F., Rodriguez-Palacios A. (2021). Regulation of Intestinal Inflammation by Dietary Fats. Front. Immunol..

[9] Svolos V., Gordon H., Lomer M.C.E., Aloi M., Bancil A., Day A.S., Day A., Fitzpatrick J.A., Gerasimidis K., Gkikas K. (2025). European Crohn’s and Colitis Organisation Consensus on Dietary Management of Inflammatory Bowel Disease. J. Crohn’s Colitis.

[10] Noejovich C.V., Miranda P., Rueda G.H., Yuan Y., Szeto J., Patel R., Zafar H., Tedesco M., Armstrong D. (2026). Understanding Dietary Beliefs, Behaviors, and Barriers in Inflammatory Bowel Disease: A Scoping Review. Clin. Nutr. ESPEN.

[11] Ribaudi E., Amato S., Becherucci G., Carillo S., Covello C., Mora V., Mentella M.C., Scaldaferri F., Gasbarrini A., Fanali C. (2025). Addressing Nutritional Knowledge Gaps in Inflammatory Bowel Disease: A Scoping Review. Nutrients.

[12] Sasson G., Hosmer C., Korzenik J. (2026). Dietary Therapies in Inflammatory Bowel Disease and Their Effects on Disease Activity and the Gut Microbiome. Nutrients.

[13] Gold S., Park S., Katz J., McClave S., Martindale R. (2025). The Evolving Guidelines on Fiber Intake for Patients with Inflammatory Bowel Disease; From Exclusion to Texture Modification. Curr. Gastroenterol. Rep..

[14] Baniqued M.R., Greb A.C., Shah N.D., Parian A.A., Li Z., Limketkai B.N. (2026). Nationwide Analysis of Food Insecurity among Individuals with Inflammatory Bowel Disease in the United States. Inflamm. Bowel Dis..

[15] Taeger M., Thiele S. (2025). How expensive is a healthy diet in Europe? Using Linear Programming as a standardised method for calculating European Food Reference Budgets. Public Health Nutr..

[16] Holko P., Kawalec P., Sajak-Szczerba M., Avedano L., Mossakowska M. (2023). Out-of-pocket expenses of patients with inflammatory bowel disease: A comparison of patient-reported outcomes across 12 European countries. Eur. J. Health Econ..

[17] Fiorino G., Fidalgo C., Adamina M., Barreiro-de Acosta M., Ali M.P., Bortlik M., Walsh A. (2025). Gaps between European Crohn’s and Colitis Organisation quality standards of care and the real world on diagnosis and monitoring inflammatory bowel disease across Europe: Results from the E-QUALITY survey. J. Crohn’s Colitis.

[18] Portmann L.J., Fitzpatrick J.A., Halmos E.P., Bryant R.V., Day A.S. (2025). Looking Beyond Nutrients, How to Assess Diet Quality in an Inflammatory Bowel Disease Population—A Narrative Review. Nutrients.

[19] Adolph T.E., Zhang J. (2022). Diet fuelling inflammatory bowel diseases: Preclinical and clinical concepts. Gut.

[20] Shivappa N., Steck S.E., Hurley T.G., Hussey J.R., Hébert J.R. (2014). Designing and Developing a Literature-Derived, Population-Based Dietary Inflammatory Index. Public Health Nutr..

[21] Lamers C.R., de Roos N.M., Witteman B.J.M. (2020). The Association between Inflammatory Potential of Diet and Disease Activity: Results from a Cross-Sectional Study in Patients with Inflammatory Bowel Disease. BMC Gastroenterol..

[22] Bakhtiari Z., Mahdavi R., Masnadi Shirazi K., Nikniaz Z. (2024). Association between Dietary Inflammatory Index and Disease Activity in Patients with Ulcerative Colitis. Sci. Rep..

[23] Rocha I.M.G.d., Torrinhas R., Fonseca D., Lyra C.d.O., de Sousa Alves Neri J.L., Balmant B.D., Callado L., Charlton K., Queiroz N., Waitzberg D.L. (2023). Pro-Inflammatory Diet Is Correlated with High Veillonella Rogosae, Gut Inflammation and Clinical Relapse of Inflammatory Bowel Disease. Nutrients.

[24] Olendzki B., Bucci V., Cawley C., Maserati R., McManus M., Olednzki E., Madziar C., Chiang D., Ward D.V., Pellish R. (2022). Dietary Manipulation of the Gut Microbiome in Inflammatory Bowel Disease Patients: Pilot Study. Gut Microbes.

[25] Bolte L.A., Vich Vila A., Imhann F., Collij V., Gacesa R., Peters V., Wijmenga C., Kurilshikov A., Campmans-Kuijpers M.J.E., Fu J. (2021). Long-Term Dietary Patterns Are Associated with pro-Inflammatory and Anti-Inflammatory Features of the Gut Microbiome. Gut.

[26] Xia X., Zhang Y., Zhu L., Ying Y., Hao W., Wang L., Huang J.Q. (2023). Liquiritin Apioside Alleviates Colonic Inflammation and Accompanying Depression-Like Symptoms in Colitis by Gut Metabolites and the Balance of Th17/Treg. Phytomedicine.

[27] Mayorga L., Noguera Segura A., Campderros L., Pons-Tarin M., Soler Z., Vega-Abellaneda S., Serrano-Gomez G., Herrera-deGuise C., Robles-Alonso V., Borruel N. (2026). Distinct Microbial Mediators Link Diet to Inflammation in Crohn’s Disease and Ulcerative Colitis. Gut.

[28] Campmans-Kuijpers M.J.E., Dijkstra G. (2021). Food and Food Groups in Inflammatory Bowel Disease (IBD): The Design of the Groningen Anti-Inflammatory Diet (GrAID). Nutrients.

[29] Keshteli A.H., Valcheva R., Nickurak C., Park H., Mandal R., van Diepen K., Kroeker K.I., van Zanten S.V., Halloran B., Wishart D.S. (2022). Anti-Inflammatory Diet Prevents Subclinical Colonic Inflammation and Alters Metabolomic Profile of Ulcerative Colitis Patients in Clinical Remission. Nutrients.

[30] Preda C.M., Istratescu D., Nitescu M., Manuc T., Manuc M., Stroie T., Tieranu C., Meianu C.G., Andrei A., Ciora C.A. (2024). Diet Optimization in Inflammatory Bowel Disease: Impact on Disease Relapse and Inflammatory Markers. A 1-Year Prospective Trial. J. Gastrointest. Liver Dis..

[31] Olendzki B.C., Silverstein T.D., Persuitte G.M., Ma Y., Baldwin K.R., Cave D. (2014). An Anti-Inflammatory Diet as Treatment for Inflammatory Bowel Disease: A Case Series Report. Nutr. J..

[32] Ratajczak A.E., Festa S., Aratari A., Papi C., Dobrowolska A., Krela-Kaźmierczak I. (2023). Should the Mediterranean Diet Be Recommended for Inflammatory Bowel Diseases Patients? A Narrative Review. Front. Nutr..

[33] Manski S., Noverati N., Policarpo T., Rubin E., Shivashankar R. (2024). Diet and Nutrition in Inflammatory Bowel Disease: A Review of the Literature. Crohns Colitis.

[34] Davis C., Bryan J., Hodgson J., Murphy K. (2015). Definition of the Mediterranean Diet: A Literature Review. Nutrients.

[35] Christensen C., Knudsen A., Arnesen E.K., Hatlebakk J.G., Sletten I.S., Fadnes L.T. (2024). Diet, Food, and Nutritional Exposures and Inflammatory Bowel Disease or Progression of Disease: An Umbrella Review. Adv. Nutr..

[36] Illescas O., Rodríguez-Sosa M., Gariboldi M. (2021). Mediterranean Diet to Prevent the Development of Colon Diseases: A Meta-Analysis of Gut Microbiota Studies. Nutrients.

[37] Turpin W., Dong M., Sasson G., Raygoza Garay J.A., Espin-Garcia O., Lee S.-H., Neustaeter A., Smith M.I., Leibovitzh H., Guttman D.S. (2022). Mediterranean-Like Dietary Pattern Associations With Gut Microbiome Composition and Subclinical Gastrointestinal Inflammation. Gastroenterology.

[38] Jaber M., Altamimi M., Altamimi A., Cavaliere S., De Filippis F. (2026). Mediterranean diet diminishes the effects of Crohn’s disease and improves its parameters: A systematic review. Nutr. Health.

[39] Singh A., Rana U., Chandnani S., Singh A., Yadav D.P., Narang H., Josy K., Ghosh T.S., Chatterjee A., Kedia S. (2026). Mediterranean Diet in Treatment of Inflammatory Bowel Disease: A Systematic Review and Meta-Analysis. Indian J. Gastroenterol..

[40] Cadoni M., Favale A., Piras R., Demurtas M., Soddu P., Usai A., Ibba I., Fantini M.C., Onali S. (2024). Adherence to Mediterranean Diet and Diet Quality in Patients with Inflammatory Bowel Disease: A Single-Center, Observational, Case-Control Study. Nutrients.

[41] Migdanis A., Migdanis I., Gkogkou N.D., Papadopoulou S.K., Giaginis C., Manouras A., Polyzou Konsta M.A., Kosti R.I., Oikonomou K.A., Argyriou K. (2024). The Relationship of Adherence to the Mediterranean Diet with Disease Activity and Quality of Life in Crohn’s Disease Patients. Medicina.

[42] Erol Doğan Ö., Karaca Çelik K.E., Baş M., Alan E.H., Çağın Y.F. (2024). Effects of Mediterranean Diet, Curcumin, and Resveratrol on Mild-to-Moderate Active Ulcerative Colitis: A Multicenter Randomized Clinical Trial. Nutrients.

[43] Haskey N., Estaki M., Ye J., Shim R.K., Singh S., Dieleman L.A., Jacobson K., Gibson D.L. (2023). A Mediterranean Diet Pattern Improves Intestinal Inflammation Concomitant with Reshaping of the Bacteriome in Ulcerative Colitis: A Randomised Controlled Trial. J. Crohn’s Colitis.

[44] Lewis J.D., Sandler R.S., Brotherton C., Brensinger C., Li H., Kappelman M.D., Daniel S.G., Bittinger K., Albenberg L., Valentine J.F. (2021). A Randomized Trial Comparing the Specific Carbohydrate Diet to a Mediterranean Diet in Adults with Crohn’s Disease. Gastroenterology.

[45] Hashash J.G., Elkins J., Lewis J.D., Binion D.G. (2024). AGA Clinical Practice Update on Diet and Nutritional Therapies in Patients With Inflammatory Bowel Disease: Expert Review. Gastroenterology.

[46] Tong T.Y.N., Imamura F., Monsivais P., Brage S., Griffin S.J., Wareham N.J., Forouhi N.G. (2018). Dietary Cost Associated with Adherence to the Mediterranean Diet, and Its Variation by Socio-Economic Factors in the UK Fenland Study. Br. J. Nutr..

[47] Rubini A., Vilaplana-Prieto C., Flor-Alemany M., Yeguas-Rosa L., Hernández-González M., Félix-García F.J., Félix-Redondo F.J., Fernández-Bergés D. (2022). Assessment of the Cost of the Mediterranean Diet in a Low-Income Region: Adherence and Relationship with Available Incomes. BMC Public Health.

[48] Colaprico C., Crispini D., Rocchi I., Kibi S., De Giusti M., La Torre G. (2024). Cost and Cost-Effectiveness of the Mediterranean Diet: An Update of a Systematic Review. Nutrients.

[49] Gold S.L., Kohler D., Freid H., Haskey N., Raman M. (2024). Food Insecurity Is Common in Patients with Inflammatory Bowel Disease and Is Associated with Increased Ultra-Processed Food Intake. Nutrients.

[50] Wang T., Masedunskas A., Willett W.C., Fontana L. (2023). Vegetarian and Vegan Diets: Benefits and Drawbacks. Eur. Heart J..

[51] Haghighatdoost F., Bellissimo N., Totosy de Zepetnek J.O., Rouhani M.H. (2017). Association of Vegetarian Diet with Inflammatory Biomarkers: A Systematic Review and Meta-Analysis of Observational Studies. Public Health Nutr..

[52] Jowett S.L., Seal C.J., Pearce M.S., Phillips E., Gregory W., Barton J.R., Welfare M.R. (2004). Influence of Dietary Factors on the Clinical Course of Ulcerative Colitis: A Prospective Cohort Study. Gut.

[53] Chiba M., Abe T., Tsuda H., Sugawara T., Tsuda S., Tozawa H., Fujiwara K., Imai H. (2010). Lifestyle-Related Disease in Crohn’s Disease: Relapse Prevention by a Semi-Vegetarian Diet. World J. Gastroenterol..

[54] Liu G.X.H., Day A.S. (2024). Plant-Based Diets for Inflammatory Bowel Disease: What Is the Evidence?. Inflamm. Bowel Dis..

[55] Peters V., Bolte L., Schuttert E.M., Andreu-Sánchez S., Dijkstra G., Weersma R.K., Campmans-Kuijpers M.J.E. (2022). Western and Carnivorous Dietary Patterns Are Associated with Greater Likelihood of IBD Development in a Large Prospective Population-Based Cohort. J. Crohn’s Colitis.

[56] Besedin D., Shah R., Brennan C., Panzeri E., Hao Van T.T., Eri R. (2024). Food Additives and Their Implication in Inflammatory Bowel Disease and Metabolic Syndrome. Clin. Nutr. ESPEN.

[57] Trakman G.L., Lin W.Y.Y., Hamilton A.L., Wilson-O’Brien A.L., Stanley A., Ching J.Y., Yu J., Mak J.W.Y., Sun Y., Niu J. (2022). Processed Food as a Risk Factor for the Development and Perpetuation of Crohn’s Disease—The ENIGMA Study. Nutrients.

[58] Epidemiology Group of the Research Committee of Inflammatory Bowel Disease in Japan (1994). Dietary and Other Risk Factors of Ulcerative Colitis. A Case-Control Study in Japan. J. Clin. Gastroenterol..

[59] Narula N., Wong E.C.L., Dehghan M., Mente A., Rangarajan S., Lanas F., Lopez-Jaramillo P., Rohatgi P., Lakshmi P.V.M., Varma R.P. (2021). Association of Ultra-Processed Food Intake with Risk of Inflammatory Bowel Disease: Prospective Cohort Study. BMJ.

[60] Hou J.K., Abraham B., El-Serag H. (2011). Dietary Intake and Risk of Developing Inflammatory Bowel Disease: A Systematic Review of the Literature. Am. J. Gastroenterol..

[61] Kempinski R., Arabasz D., Neubauer K. (2024). Effects of Milk and Dairy on the Risk and Course of Inflammatory Bowel Disease versus Patients’ Dietary Beliefs and Practices: A Systematic Review. Nutrients.

[62] Constantine-Cooke N., Gros B., Plevris N., Williams L.J., Jones G.R., Kyle J., Kennedy N.A., Velasco-Pardo V., Rudge A., Alexander D. (2026). Associations between Demographic, Clinical and Dietary Factors and Flares in Inflammatory Bowel Disease: The PREdiCCt Prospective Cohort Study. Gut.

[63] Wei Y., Li J., Li J., Liu C., Guo X., Liu Z., Zhang L., Bao S., Wu X., Su W. (2024). Dietary Long-Chain Fatty Acids Promote Colitis by Regulating Palmitoylation of STAT3 through CD36-Mediated Endocytosis. Cell Death Dis..

[64] Shon W.-J., Jung M.H., Kim Y., Kang G.H., Choi E.Y., Shin D.-M. (2023). Sugar-Sweetened Beverages Exacerbate High-Fat Diet-Induced Inflammatory Bowel Disease by Altering the Gut Microbiome. J. Nutr. Biochem..

[65] Martin O.C.B., Olier M., Ellero-Simatos S., Naud N., Dupuy J., Huc L., Pierre F.H. (2019). Haem iron reshapes colonic luminal environment: Impact on mucosal homeostasis and microbiome through aldehyde formation. Microbiome.

[66] Komisarska P., Pinyosinwat A., Saleem M., Szczuko M. (2024). Carrageenan as a Potential Factor of Inflammatory Bowel Diseases. Nutrients.

[67] Martino J.V., Van Limbergen J., Cahill L.E. (2017). The Role of Carrageenan and Carboxymethylcellulose in the Development of Intestinal Inflammation. Front. Pediatr..

[68] Bhattacharyya S., Shumard T., Xie H., Dodda A., Varady K.A., Feferman L., Halline A.G., Goldstein J.L., Hanauer S.B., Tobacman J.K. (2017). A Randomized Trial of the Effects of the No-Carrageenan Diet on Ulcerative Colitis Disease Activity. Nutr. Healthy Aging.

[69] Sandall A.M., Cox S.R., Lindsay J.O., Gewirtz A.T., Chassaing B., Rossi M., Whelan K. (2020). Emulsifiers Impact Colonic Length in Mice and Emulsifier Restriction Is Feasible in People with Crohn’s Disease. Nutrients.

[70] Wellens J., Vanderstappen J., Hoekx S., Vissers E., Luppens M., Van Elst L., Lenfant M., Raes J., Derrien M., Verstockt B. (2026). Effect of Five Dietary Emulsifiers on Inflammation, Permeability, and the Gut Microbiome: A Placebo-Controlled Randomized Trial. Clin. Gastroenterol. Hepatol..

[71] Vissers E., Wellens J., Sabino J. (2022). Ultra-Processed Foods as a Possible Culprit for the Rising Prevalence of Inflammatory Bowel Diseases. Front. Med..

[72] Hrncirova L., Machova V., Trckova E., Krejsek J., Hrncir T. (2019). Food Preservatives Induce Proteobacteria Dysbiosis in Human-Microbiota Associated Nod2-Deficient Mice. Microorganisms.

[73] Raoul P., Cintoni M., Palombaro M., Basso L., Rinninella E., Gasbarrini A., Mele M.C. (2022). Food Additives, a Key Environmental Factor in the Development of IBD through Gut Dysbiosis. Microorganisms.

[74] Kuang R., O’Keefe S.J.D., Ramos Del Aguila de Rivers C., Koutroumpakis F., Binion D.G. (2023). Is Salt at Fault? Dietary Salt Consumption and Inflammatory Bowel Disease. Inflamm. Bowel Dis..

[75] Barbalho S.M., Goulart R.d.A., Aranão A.L.d.C., de Oliveira P.G.C. (2018). Inflammatory Bowel Diseases and Fermentable Oligosaccharides, Disaccharides, Monosaccharides, and Polyols: An Overview. J. Med. Food.

[76] McGaugh E., Barthel B. (2022). A Review of Ketogenic Diet and Lifestyle. Mo. Med..

[77] Guevara M., Salamanca-Fernández E., Miqueleiz E., Gavrila D., Amiano P., Bonet C., Rodríguez-Barranco M., Huerta J.M., Bujanda L., Sánchez M.J. (2021). Inflammatory Potential of the Diet and Incidence of Crohn’s Disease and Ulcerative Colitis in the EPIC-Spain Cohort. Nutrients.

[78] Yan D., Ye S., He Y., Wang S., Xiao Y., Xiang X., Deng M., Luo W., Chen X., Wang X. (2023). Fatty Acids and Lipid Mediators in Inflammatory Bowel Disease: From Mechanism to Treatment. Front. Immunol..

[79] Salter A.M. (2018). The Effects of Meat Consumption on Global Health. Rev. Sci. Tech..

[80] Kostovcikova K., Coufal S., Galanova N., Fajstova A., Hudcovic T., Kostovcik M., Prochazkova P., Jiraskova Zakostelska Z., Cermakova M., Sediva B. (2019). Diet Rich in Animal Protein Promotes Pro-Inflammatory Macrophage Response and Exacerbates Colitis in Mice. Front. Immunol..

[81] Norwitz N.G., Soto-Mota A. (2024). Case Report: Carnivore-Ketogenic Diet for the Treatment of Inflammatory Bowel Disease: A Case Series of 10 Patients. Front. Nutr..

[82] Cox S.R., Lindsay J.O., Fromentin S., Stagg A.J., McCarthy N.E., Galleron N., Ibrahim S.B., Roume H., Levenez F., Pons N. (2020). Effects of Low-FODMAP Diet on Symptoms, Faecal Microbiome, and Markers of Inflammation in Patients with Quiescent Inflammatory Bowel Disease in a Randomized Trial. Gastroenterology.

[83] Wang C., Wu R., Yao D., Yu Z., Shen X. (2024). Comparison of Dietary Inflammatory Index and Inflammatory Biomarkers between Vegetarians and Omnivores in Chinese Population. Sci. Rep..

[84] Tian Z., Zhuang X., Zhao M., Zhuo S., Li X., Ma R., Li N., Liu C., Zhu Y., Tang C. (2021). Index-Based Dietary Patterns and Inflammatory Bowel Disease: A Systematic Review of Observational Studies. Adv. Nutr..

